# Modified task-based learning program promotes problem-solving capacity among Chinese medical postgraduates: a mixed quantitative survey

**DOI:** 10.1186/s12909-017-0994-0

**Published:** 2017-09-07

**Authors:** Yanping Tian, Chengren Li, Jiali Wang, Qiyan Cai, Hanzhi Wang, Xingshu Chen, Yunlai Liu, Feng Mei, Lan Xiao, Rui Jian, Hongli Li

**Affiliations:** Department of Histology and Embryology, Third Military Medical University, 30# Gaotanyan St., Shapingba District, Chongqing, 400038 China

**Keywords:** Modified task-based learning program, Medical postgraduate education, Problem-solving capacity, Immunohistochemistry

## Abstract

**Background:**

Despite great advances, China’s postgraduate education faces many problems, for example traditional lecture-based learning (LBL) method provides fewer oppotunities to apply knowledge in a working situation. Task-based learning (TBL) is an efficient strategy for increasing the connections among skills, knowledge and competences. This study aimed to evaluate the effect of a modified TBL model on problem-solving abilities among postgraduate medical students in China.

**Methods:**

We allocated 228 first-year postgraduate students at Third Military Medical University into two groups: the TBL group and LBL group. The TBL group was taught using a TBL program for immunohistochemistry. The curriculum consisted of five phases: task design, self-learning, experimental operations, discussion and summary. The LBL group was taught using traditional LBL. After the course, learning performance was assessed using theoretical and practical tests. The students’ preferences and satisfaction of TBL and LBL were also evaluated using questionnaires.

**Results:**

There were notable differences in the mean score rates in the practical test (*P* < 0.05): the number of high scores (>80) in the TBL group was higher than that in the LBL group. We observed no substantial differences in the theoretical test between the two groups (*P* > 0.05). The questionnaire results indicated that the TBL students were satisfied with teaching content, teaching methods and experiment content. The TBL program was also beneficial for the postgraduates in completing their research projects. Furthermore, the TBL students reported positive effects in terms of innovative thinking, collaboration, and communication.

**Conclusions:**

TBL is a powerful educational strategy for postgraduate education in China. Our modified TBL imparted basic knowledge to the students and also engaged them more effectively in applying knowledge to solve real-world issues. In conclusion, our TBL established a good foundation for the students’ future in both medical research and clinical work.

**Electronic supplementary material:**

The online version of this article (10.1186/s12909-017-0994-0) contains supplementary material, which is available to authorized users.

## Background

Medical education is a continuous, lifelong process and postgraduate medical education (PGME) is an important part of that process. PGME is a key element in the training of medical professionals and developing their innovative ability [1]. In China, postgraduate education started in 1949. As of 2014, it had produced 2 million enrolled graduate students with about 70,000 doctoral degree recipients and 500,000 master’s degree recipients each year [2]. With this large number of postgraduate students, China’s postgraduate educational system faces many problems [2, 3]. Policy reforms with respect to faculty development, quality standardization, curriculum reform and accreditation are currently being implemented to adapt the challenges of rapidly changing, globalized education [4, 5].

At Third Military Medical University, PGME is a 3-year program. The 1st year is devoted to basic knowledge. The subsequent 2 years focus on research or clinical work and students who wish to obtain a master’s degree must publish an article in an academic journal [6, 7]. In our university, postgraduate courses consist of core and elective curricula. Immunohistochemistry is an elective curriculum in early PGME and widely applied in medical research and clinical diagnosis [8]. Many postgraduates use immunohistochemical techniques to complete their research as part of their postgraduate degree work. In China, many university tutors teach immunohistochemistry using traditional lecture-based learning (LBL). LBL is a good method for imparting basic knowledge to students. Taught that way, many students acquire knowledge about immunohistochemistry but lack the ability to solve real associated problems. It often leads to unsatisfactory learning outcomes: students passively receive knowledge from instructors without having the motivation to study and innovate [9, 10]. Developing key skills and innovation, such as problem-solving skills, is an important object in PGME [11, 12].

To meet these challenges, reforms in educational strategies have been recommended in the curricula of Chinese medical schools over the past 2 decades. One such strategy is that of a task-based learning (TBL) approach, in which learning takes place with respect to many tasks assigned by instructors [13, 14]. TBL is similar to problem-based learning (PBL), but there are essential differences in strategy. In contrast to PBL, TBL focuses on a set of tasks; it offers practical advantages, saves resources, and increases the connections among skills, knowledge, and competences [15]. TBL has been shown to be an effective, efficient strategy in education [15]. In undergraduate education for health professionals, TBL supports the integration of medical knowledge with patient care by providing a context for learning and developing transferable skills [16].

The aim of the present study was to describe and evaluate TBL in teaching immunohistochemistry to postgraduates in China. Information gained from this study can help us understand our modified TBL in imparting basic knowledge to students and engage them more effectively in applying knowledge to solve real-world issues. It can also help them develop skills in collaboration and communication, thereby establishing a good foundation for basic medicine and clinical medical research work in the future.

## Methods

### Participant sampling

The participating students were 228 first-year postgraduates at Third Military Medical University (Chongqing, China); they were randomly allocated into two groups of 114: the TBL group and LBL group. Table 1 shows the basic characteristics of the two groups. No significant differences were evident between the two groups in terms of student numbers, sex, or age (*P* > 0.05).

**Table 1:** Basic characteristics of participants

Groups	TBL group (n = 114) Number(%)	LBL group (n = 114) Number(%)
Male	83 (72.8)	84 (73.7)
Female	31 (27.2)	30 (26.3)
Specialty		
Clinical, academic	81	82
Research, academic	33	32
Mean (SD)		
Mean age (years)	24.3 (1.5)	24.2 (1.7)

### Teaching methods

#### TBL group

**Fig. 1:**
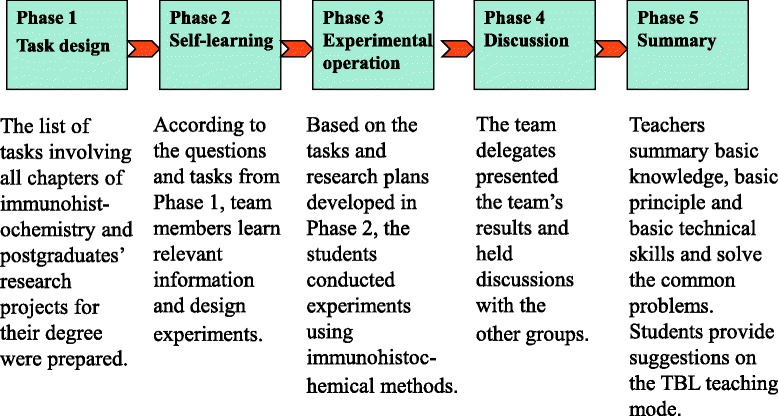
Conceptual framework for the TBL model

The descriptive data of the TBL group appear in Table 1. This group of 114 students was randomly divided into 30 smaller groups, each composed of three to four participants. The TBL model consisted of five phases. Figure 1 presents the conceptual framework of that model.

##### Phase 1: Task design

The tasks consisted of two parts. In the first part, a list of tasks covering all chapters of an immunohistochemistry textbook was prepared. The list was systematically clustered into three main groups: experimental methods, experimental tools and quantification of morphometric analysis. Further details of the tasks (for example, hematoxylin and eosin staining) appear in Table 2. In the second part, the task was the students’ research project for their degree; it involved a typical medical case that demanded the use of immunohistochemical techniques (Table 3). A study guide explained the learning issues involved with each task.

**Table 2:** Tasks in the TBL model for immunohistochemistry and the task of hematoxylin and eosin staining as an example

Principle tasks	Learning objectives
Experimental methods	Paraffin section technique; Frozen section technique
	HE staining; Silver staining
	Nissl staining; Giemsa staining
	Oil red O dyeing; Hoechst staining
	Tissue microarrays; In situ hybridization
	Periodic acid–Schiff (PAS) reaction
	Immunoperoxidase immunohistochemistry
	Immunofluorescence histochemistry
Experimental tools	Confocal microscopy; Flow cytometer
	Scanning electron microscope (SEM)
	Transmission electron microscopy (TEM)
	Immunoelectron microscopy
Quantification of morphometric analysis	Quantification of morphological images
	Stereology
Questions	
1. What is your research project? Do you use hematoxylin-eosin staining (HE staining) in your research work? (Phase 1)	
2. What is the principle of HE staining? (Phase 1)	
3. What problems HE staining can solve? (Phase 1)	
4. What reagents are needed in this experiment? How to configure these buffer solutions? (Phase 3)	
5. What is the experimental protocol of HE staining? (Phase 3)	
6. How observed the experimental results of HE staining? (Phase 3)	
7. There are some key options and considerations to take into account.(Teachers provided, Phase 4)	
8. What are the advantages and disadvantages of HE staining compared with other methods? (Teachers provided, Phase 5)

**Table 3:** Case materials and guiding questions

Case	Questions
Embryonic stem cells (ES cells) are pluripotent stem cells derived from the inner cell mass of blastocyst. ES cells are able to differentiate to generate primitive ectoderm, which ultimately differentiates into all derivatives of the three primary germ layers: ectoderm, endoderm, and mesoderm, which differentiate more than 220 cell types in the adult body. Because of their plasticity and potentially unlimited capacity for self-renewal, ES cell therapies have been proposed for regenerative medicine and tissue replacement after injury or disease. They are also models for drug screening, gene research and so on.	1. How to observe the development structures of three germ layers? (HE staining)
	2. How many neuroectoderm cells were obtained in ES cell differentiation? (flow cytometry)
	3. How to observe characteristic protein expression of neuroectoderm? (Immunoperoxidase immunohistochemistry, Immunofluorescence histochemistry, In situ hybridization)
	4. How to observe the microstructure of neurons? (Electron microscope, Immunoelectron microscopy)
	5. How to quantify the morphological images? (Stereology)

##### Phase 2: Self-learning

According to the questions and tasks from Phase 1, the team members made learning plans and acquired relevant information from a range of resources, including textbooks, libraries and the Internet. Through self-study, discussion, analysis and summary, the group members developed opinions and a scheme for their experiment. The team members collaborated with one another and teachers provided assistance throughout the whole process.

##### Phase 3: Experimental operations

Based on the tasks and research plans developed in Phase 2, the students conducted experiments according to an experimental scheme of their own design using immunohistochemical methods. In the course of the experiment, teachers provided the guidance. The students were graded based on the results of those experiments.

##### Phase 4: Discussion

After the self-learning and experimental operations, team delegates presented their results and held discussions with the other groups. All the students participated in the discussions.

##### Phase 5: Summary

The teachers gave a synopsis of the basic knowledge, basic principles and technical skills covered in the course. Teachers also summarized key and difficult points of the course and addressed common problems encountered by the students. The students participated in the meetings of this phase and provided suggestions about the TBL teaching mode.

#### LBL group

The descriptive data of the LBL group appear in Table 1. The 114 students in this group received the same theory by traditional LBL, which was undertaken by the same staff as with the TBL group. After theory learning, the students conducted experimental operations, in which the teachers provided the experimental materials and protocols. Open classroom discussions took place, but the tasks did not constitute the students’ research project for their degree.

### Evaluation methods

We used three means of evaluating the study.

1. **Written immunohistochemistry examination (theoretical test).** Students in both the TBL and LBL groups took a final examination after finishing the course. The examination included basic knowledge, basic principles and technical skills related to immunohistochemistry. The scoring staffs were blinded to the identity of the students and their assigned group.
2. **Examination on experimental operations (practical test).** The results of the experimental operations involving immunohistochemical techniques (Phase 3) were collected and evaluated.
3. **Questionnaire survey.** After the end of the course, the students completed a questionnaire, which evaluated their satisfaction with the course. Students in both the TBL and LBL groups filled out the questionnaire to rate the course. Satisfaction was evaluated by means of a four-point scale: excellent, good, fair and poor.

### Statistical analyses

We summarized the data from the students’ evaluation ratings using descriptive statistics (means, standard deviation [SD], and response rates). We conducted statistical analysis using SPSS 17.0 software for Windows (SPSS Inc., Chicago, IL, USA). Data are presented as means ± SD. Statistical analysis between the groups was evaluated using *t* tests and analysis of variance (ANOVA); a *P* value <0.05 was considered significant.

## Results

### Participation

As evident in Table 1, there were no significant differences between the groups in terms of student numbers, sex, age, or specialty (*P* > 0.05). All the students originally included in this study took the final examination in immunohistochemistry. Before and after the course, all the students completed the questionnaire.

### Immunohistochemistry examination results of the two groups

**Fig. 2:**
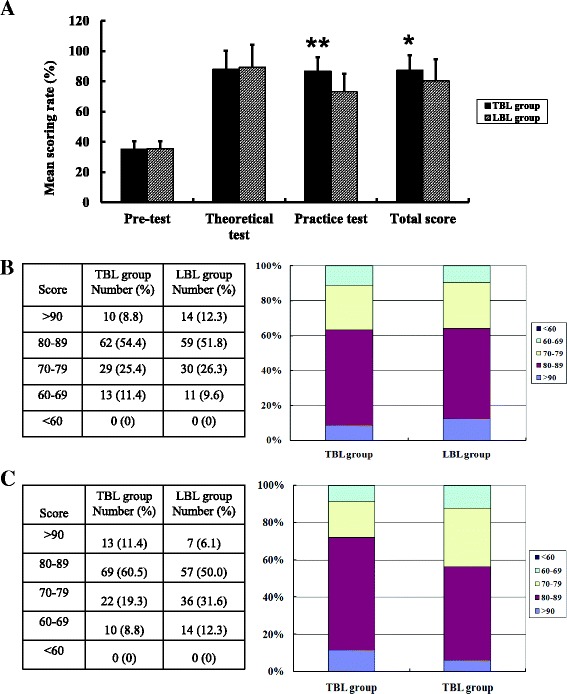
Results of the final examination. **a**. Mean examination scores (X ± SD) of the TBL group (*n* = 114) and LBL group (n = 114). *P < 0.05, **P < 0.01. **b**. Demographic characteristics for the theoretical test. **c**. Demographic characteristics for the practical test

To determine the extent of the students’ knowledge acquisition, we gave a pre-test questionnaire before the course (Details in Additional file 1) and a post-test afterward; we analyzed the differences between the test scores. An analysis of the test scores appears in Fig. 2. We found no difference in the students’ pre-test scores. However, the post-test results indicated that the total scores of the TBL group (mean ± SD, 87.3 ± 9.9) were significantly higher than those of the LBL group (80.3 ± 14.3; *P* < 0.05; Figure 2A). Further analysis of the mean scores of the practical test revealed significant differences: 86.7 ± 9.1 in the TBL group versus 73.3 ± 11.5 in the LBL group; *P* < 0.01. There were no substantial differences between the two groups in the theoretical test (*P* > 0.05). We analyzed the detailed distribution of the scores based on the score for knowledge acquisition. We found that there was no significant difference between the two groups in the theoretical test (Fig. 2b; *P* > 0.05). However, the TBL group achieved the highest scores in the practical test: the number of high scores (>80) in the TBL group was higher than that in the LBL group (Fig. 2c; *P* < 0.01).

### Analysis of questionnaire results

After the course, the students were asked to complete an anonymous questionnaire. The contents of the questionnaire and the results are shown in Tables 4 and 5. In all, 84.2% of the students were positive (“excellent” or “good” on the four-point scale) with respect to course satisfaction for TBL; that figure was 79% for LBL. Students in the TBL course showed higher satisfaction in terms of curriculum, teaching content, teaching method, experiment content and experiment course than those in the LBL group (Table 4).

**Table 4:** Questionnaire results about the teaching mode among students in the TBL and LBL groups

	TBL group (N = 114) Number (%)				LBL group (N = 114) Number (%)			
	Excellent	Good	Fair	Poor	Excellent	Good	Fair	Poor
The whole course	56(49.1)	40(35.1)	10(8.8)	8(7.0)	42(36.9)	48(42.1)	20(17.5)	4(3.5)
The content of courses	58(50.9)	42(36.8)	12(10.5)	2(1.8)	53(46.4)	40(35.1)	19(16.7)	2(1.8)
The teaching method	73(64.0)	28(24.6)	13(11.4)	0(0)	33(28.9)	62(54.4)	18(15.8)	1(0.9)
The experimental course content	45(39.4)	52(45.6)	15(13.2)	2(1.8)	45(39.5)	46(40.3)	18(15.8)	5(4.4)
The form of experiment course	82(71.9)	23(20.2)	8(7.0)	1(0.9)	42(36.8)	55(48.3)	12(10.5)	5(4.4)

**Table 5:** Questionnaire results about the students’ research projects in the TBL and LBL groups

	TBL group (N = 114) Number (%)				LBL group (N = 114) Number (%)			
	Excellent	Good	Fair	Poor	Excellent	Good	Fair	Poor
Helpful to complete the project for master degree	61(53.5)	38(33.3)	14(12.3)	1(0.9)	23(20.2)	43(37.7)	40(35.1)	8(7.0)
Improve ability of project design	53(46.5)	45(39.5)	14(12.3)	2(1.7)	15(13.2)	51(44.7)	40(35.1)	8(7.0)
Searching related literatures	63(55.3)	34(29.8)	13(11.4)	4(3.5)	24(21.1)	34(29.8)	50(43.9)	6(5.2)
Mastering related experimental methods	67(58.8)	37(32.5)	10(8.7)	0(0)	23(20.2)	57(50.0)	31(27.2)	3(2.6)
Team members are cooperated to complete tasks.	32(28.1)	61(53.5)	19(16.7)	2(1.7)	15(13.2)	35(30.7)	59(51.7)	5(4.4)
Teachers play a better role for guiding	45(39.5)	56(49.1)	12(10.5)	1(0.9)	45(39.5)	37(32.5)	30(26.3)	2(1.7)

In addition to mastering the theory and techniques of immunohistochemistry, an important function of TBL in the present study involved solving problems related to experiments using immunohistochemical techniques. The questionnaire results showed that the TBL model was beneficial in helping the students complete their research projects. The TBL students were able to develop their experimental design skills. They also gained the ability to find relevant documents and acquire immunohistochemical techniques. The TBL model promoted the team members to cooperate in completing their tasks. With respect to the research projects, the LBL students’ satisfaction was lower than that of the TBL students (Table 5).

The students regarded TBL as an innovative learning method and agreed that it created an active classroom atmosphere in the immunohistochemistry course (Table 6). Compared with the traditional method, the TBL model enhanced learning motivation and self-learning ability, and learning efficiency; it reinforced problem-solving ability and improved students’ ability in cooperation and communication. Thus, the TBL method will probably be welcomed by most students.

**Table 6:** Questionnaire results about teaching effects in the TBL and LBL groups

	TBL group (N = 114) Number (%)				LBL group (N = 114) Number (%)			
	Excellent	Good	Fair	Poor	Excellent	Good	Fair	Poor
Activating class atmosphere	96(84.2)	18(15.8)	0(0)	0(0)	23(20.2)	21(18.4)	54(47.4)	16(14.0)
Stimulating learning interest	54(47.4)	44(38.6)	16(14.0)	0(0)	23(20.2)	39(34.2)	34(29.9)	28(24.7)
Improving self-learning ability	63(55.2)	35(30.7)	15(13.2)	1(0.9)	19(16.6)	52(45.6)	32(28.1)	10(8.7)
Improving learning efficiency	39(34.2)	54(47.4)	20(17.5)	1(0.9)	32(28.1)	33(28.9)	42(36.9)	7(6.1)
Enhancing extra-curricular knowledge acquisition ability	83(72.8)	23(20.2)	8(7.0)	0(0)	30(26.3)	65(57.0)	18(15.8)	1(0.9)
Increasing motivation and active thinking	75(65.8)	33(28.9)	6(5.3)	0(0)	21(18.4)	53(46.5)	24(21.1)	16(14.0)
Developing problem solving skills	72(63.1)	32(28.1)	9(7.9)	1(0.9)	27(23.7)	61(53.5)	22(19.3)	4(3.5)
Promoting teamwork	48(42.1)	54(47.4)	11(9.6)	1(0.9)	28(24.6)	45(39.5)	34(29.9)	7(6.0)
Improving communication ability	53(46.5)	47(41.2)	14(12.3)	0(0)	29(25.4)	39(34.2)	31(27.2)	15(13.2)

## Discussion

Postgraduates possess basic medical knowledge and considerable clinical experience. It is important for postgraduates to improve their problem-solving ability through practical training [17]. TBL can clearly help make a curriculum more relevant to professional practice; as a training approach, TBL can be advocated for enhancing the connections among skills, knowledge and competences [18]. In the present study, the TBL model was used to improve students’ problem-solving ability, collaboration, and communication.

As an experimental technology curriculum, the teaching model for immunohistochemistry in many China’s universities is still a traditional LBL program. The LBL model can impart knowledge to students systematically and comprehensively, but it is insufficient for solving related problems [10]. In LBL classes, many students have difficulty in linking theory with laboratory exercises. Students blindly follow step-by-step protocols without having proper opportunity to think critically about the task at hand [9]. TBL is a useful method for medical students in that it helps them consider the connections among skills, knowledge, and competences. Our results indicate that the mean theoretical test scores of the TBL group did not significantly differ from those of the LBL group; however, the TBL group achieved the highest scores in the practical test. This finding suggests that our modified TBL imparts knowledge to students and promotes the translation of knowledge into actual practice.

LBL is a good method for imparting basic knowledge to students; however, students’ learning initiative is not reinforced owing to the lack of knowledge in solving real problems [10]. The LBL model has the problem of adapting medical education to real challenges; one such challenge is a disparity between a physician’s responsibilities to a patient and research demands. It is necessary for medical education to be designed so as to overcome discipline boundaries [19, 20]. TBL supports “education for capability.” Students’ learning is directed toward mastering the competencies relating to the tasks at hand. Publication of research papers is a priority for Chinese postgraduates [21]. Our results show that the TBL model is beneficial for completing research projects. The students found that TBL could improve their project design skills, help them find relevant papers and acquire immunohistochemical techniques. TBL engages students more effectively than LBL in applying knowledge in solving real-world problems.

Innovative interdisciplinary collaborations are needed to train medical workers and researchers [22, 23]. Collaborative approaches to education and training are increasingly expected both among and within institutions. TBL can provide a flexible framework to support collaboration, which motivates students to learn and promotes team collaboration [13]. With TBL in the present study, the students worked collaboratively with one another, developed task-related experimental plans and attempted to understand both the tasks themselves and the concepts and mechanisms underlying those tasks. The TBL approach can help break down the barriers that separate different areas of education and training as well as bridge gaps between classmates and teachers.

Communication with patients is an important skill for physicians. Attitudes toward developing communication skills tend to improve as medical students graduate and enter postgraduate training programs [24]. In their postgraduate training, students gradually acquire communication skills in the learning program [25]. The TBL model employed in the present study helped students develop communication skills, including communicating with teachers and classmates.

However, some participants in this study were negative about TBL. Their comments focused mainly on the teaching topics being less structured and less systematically organized than with LBL. Accordingly, it was more difficult for those students to gain a comprehensive understanding of the subject matter. TBL is less effective than LBL among students with poor self-directed study abilities.

In conclusion, our modified TBL imparted basic knowledge to students and also engaged them more effectively in applying knowledge to solve real-world issues; it helped them develop skills related to problem-solving ability, collaboration and communication. Our TBL established a good foundation for the students’ futures in both medical research and clinical work.

## Conclusions

Our findings indicate that our modified TBL is a powerful educational strategy and that it is effective for Chinese postgraduate education. Our modified TBL imparted basic knowledge to students and also engaged them more effectively in applying knowledge to solve real-world issues; it helped them develop skills related to problem-solving ability, collaboration, and communication. In conclusion, our TBL established a good foundation for the students’ futures in both medical research and clinical work.

## Additional file

Additional file 1: Pre-text questionnaire about immunohistochemistry. (DOCX 16 kb)

## Abbreviations

LBL: Lecture-based learning
PBL: Problem-based learning
PGME: Postgraduate medical education
TBL: Task-based learning
