# Supplementary material for: Modified task-based learning program promotes problem-solving capacity among Chinese medical postgraduates: a mixed quantitative survey
Source: BMC Med Educ. 2017 Sep 7;17:153. doi: 10.1186/s12909-017-0994-0 (PMC5590117; doi:10.1186/s12909-017-0994-0)
Supplement: Additional file 1: — Pre-text questionnaire about immunohistochemistry. (DOCX 16 kb) [file 12909_2017_994_MOESM1_ESM.docx]

Pre-text questionnaire about immunohistochemistry

Explaination:

(1) Experimental principle or Working principle;

(2) Experimental methods and procedures or operating procedure;

(3) Experiment and observation by self

A: According with 3/3; Very familiar

B: According with 2/3; Familiar

C: According with 1/3; Poor familiar

D: According with 0/3; No familiar

1. The paraffin section technique is one of techniques used commonly in immunohistochemistry. Are you familiar with paraffin section technique?

A. Very familiar; B. Familiar; C. Poor familiar; D. No familiar

2. Frozen section technique is a method that makes the microstructure quickly cool and then carries out slicing. The frozen section technique is also the most common technique in immunohistochemistry techniques. Are you familiar with frozen section technique?

A. Very familiar; B. Familiar; C. Poor familiar; D. No familiar

3. HE staining is one of the principal stains in immunohistochemistry. It is the most widely used stain in medical diagnosis. Are you familiar with HE staining?

A. Very familiar; B. Familiar; C. Poor familiar; D. No familiar

4. Silver staining is the use of silver to selectively alter the appearance of a target in microscopy of histological sections. It aids the visualization of targets of interest, namely intracellular and extracellular components such as DNA and proteins. Are you familiar with silver staining?

A. Very familiar; B. Familiar; C. Poor familiar; D. No familiar

5. Nissl staining method is useful to localize the cell body, as it can be seen in the soma and dendrites of neurons. Are you familiar with Nissl staining?

A. Very familiar; B. Familiar; C. Poor familiar; D. No familiar

6. Giemsa staining is a classic blood film stain for peripheral blood smears and bone marrow specimens. Are you familiar with Giemsa staining?

A. Very familiar; B. Familiar; C. Poor familiar; D. No familiar

7. Oil red O is a lysochrome (fat-soluble dye) diazo dye used for staining of neutral triglycerides and lipids on frozen sections and some lipoproteins on paraffin sections. Are you familiar with Oil red O dyeing?

A. Very familiar; B. Familiar; C. Poor familiar; D. No familiar

8. Hoechst staining is a part of a family of blue fluorescent dyes used to stain DNA. Are you familiar with Hoechst staining?

A. Very familiar; B. Familiar; C. Poor familiar; D. No familiar

9. Periodic acid–Schiff (PAS) is a staining method used to detect polysaccharides such as glycogen, and mucosubstances such as glycoproteins, glycolipids and mucins in tissues. Are you familiar with PAS reaction?

A. Very familiar; B. Familiar; C. Poor familiar; D. No familiar

10. Immunoperoxidase immunohistochemistry is used enzyme labeled specific antibody or antigen in tissues and cells *in situ* to analyze the corresponding antigen or antibody qualitative and positioning and quantitative determination by colour reaction of antigen antibody reaction and immunohistochemistry. Are you familiar with immunoperoxidase histochemical stain?

A. Very familiar; B. Familiar; C. Poor familiar; D. No familiar

11. Immunofluorescence histochemistry is based on the principle of antigen antibody reaction, the antigen or antibody marker known on fluorescein, using the fluorescent antibody (or antigen) as corresponding antigen probe cells or tissues (or antibody). Are you familiar with immunofluorescence histochemical stain?

A. Very familiar; B. Familiar; C. Poor familiar; D. No familiar

12. Tissue microarrays (also TMAs) consist of paraffin blocks in which separate tissue cores are assembled in array fashion to allow multiplex histological analysis. Are you familiar with tissue microarray technology?

A. Very familiar; B. Familiar; C. Poor familiar; D. No familiar

13. *In situ* hybridization (ISH) is a type of hybridization that uses a labeled complementary DNA, RNA or modified nucleic acids strand (i.e., probe) to localize a specific DNA or RNA sequence in a portion or section of tissue (in situ). Are you familiar with *in situ* hybridization?

A. Very familiar; B. Familiar; C. Poor familiar; D. No familiar

14. *In situ* PCR is the PCR reaction in tissue cells, it combines the advantages with cellular localization ability *in situ* hybridization and highly specific and sensitive PCR technology, is a great potential for new technology research and clinical diagnosis of cytology in the field. Are you familiar with in situ PCR Technology?

A. Very familiar; B. Familiar; C. Poor familiar; D. No familiar

15. Confocal microscopy, most frequently confocal laser scanning microscopy (CLSM), is an optical imaging technique for increasing optical resolution and contrast of a micrograph by means of adding a spatial pinhole placed at the confocal plane of the lens to eliminate out-of-focus light. Are you familiar with confocal laser scanning microscope?

A. Very familiar; B. Familiar; C. Poor familiar; D. No familiar

16. A scanning electron microscope (SEM) is a type of electron microscope that produces images of a sample by scanning it with a focused beam of electrons. Are you familiar with scanning electron microscope?

A. Very familiar; B. Familiar; C. Poor familiar; D. No familiar

17. Transmission electron microscopy (TEM) is a microscopy technique in which a beam of electrons is transmitted through an ultra-thin specimen, interacting with the specimen as it passes through it. Are you familiar with transmission electron microscope?

A. Very familiar; B. Familiar; C. Poor familiar; D. No familiar

18. Immunoelectron microscopy is a product of the combination of immunohistochemistry and electron microscopy. It is a methodology for the study of the ultrastructure and the localization of antigen and antibody. Are you familiar with immunoelectron microscopy?

A. Very familiar; B. Familiar; C. Poor familiar; D. No familiar

19. In biotechnology, flow cytometry is a laser- or impedance-based, biophysical technology employed in cell counting, cell sorting, biomarker detection and protein engineering, by suspending cells in a stream of fluid and passing them by an electronic detection apparatus. Are you familiar with flow cytometry?

A. Very familiar; B. Familiar; C. Poor familiar; D. No familiar

20. Stereo is a method of sound reproduction that creates an illusion of multi-directional audible perspective. Are you familiar with stereo technology?

A. Very familiar; B. Familiar; C. Poor familiar; D. No familiar
